# Gamma irradiation-enhanced performance of waste LLDPE thermally transformed into advanced sponge-like material for oil decontamination

**DOI:** 10.1038/s41598-023-46194-w

**Published:** 2023-11-06

**Authors:** H. M. Gayed, Mohamed Mohamady Ghobashy

**Affiliations:** Radiation Research of Polymer Chemistry Department, National Center for Radiation Research and Technology (NCRRT), Egyptian Atomic Energy Authority (EAEA), Cairo, Egypt

**Keywords:** Environmental sciences, Chemistry, Materials science

## Abstract

In this study, the development of advanced materials for the removal of oil–water pollution was explored, with a focus on environmental protection. The primary novelty of this research involved the conversion of waste Linear low-density polyethylene (LLDPE) into a sponge-like material denoted as sLLDPE. The process of converting involved thermal treatment in castor oil, resulting in the creation of a porous structure within the material. This sLLDPE material exhibited remarkable oil adsorbent properties and demonstrated enhanced performance in the removal of various organic contaminants from both aqueous and oil-based systems. Furthermore, gamma irradiation-induced crosslinking reactions were implemented within a dose range of 0 up to 90 kGy to further improve its oil removal capabilities. Comparing samples subjected to a radiation dose of 50 kGy with those receiving no irradiation (0 kGy), it was observed that the maximum adsorption capacities for various oils, including crude oil, gasoline oil, motor oil, pump oil, and waste oil, increased significantly. Specifically, the adsorption capacities increased by approximately 216.2%, 235.3%, 24.1%, 111.5%, and 18.6% for the respective oils. It rapidly separated oil–water mixtures with ~ 100% efficiency in a column system and maintained performance over 20 reuse cycles. The converted sLLDPE sponge exhibited excellent organics removal across solvents. The findings of this study not only shed light on the impact of irradiation on polymeric materials but also contribute to our understanding of their potential applications in environmental cleanup processes.

## Introduction

Oil–water separation was crucial for global water pollution, especially with organic pollutants^1^. Water source contamination by organics raises environmental alarms, demanding solutions^2^. In the realm of oil–water separation, adsorption stood as a cost-effective method for the removal of organic pollutants^3^, yielding high-quality treated water. Activated carbon, a widely employed adsorbent, owes its effectiveness to its vast surface area^4^. This porous carbon material is typically produced by heating carbon sources without oxygen^5^. Zeolite, a porous aluminosilicate mineral^6^, offers selective adsorption capabilities targeting organics, metals, and ammonia. Complementing these traditional materials are newer alternatives like activated alumina, aerogels, and polymer-based resins, each with distinct properties and surfaces tailored for diverse organic pollutants.

In general, the use of LLDPE nanocomposites for oil removal is an active area of research^7^. Also, (LLDPE) coated polyvinyl alcohol (PVA) nanofibrous membrane to make it superhydrophobic and Superoleophilic^8^. A novel approach using electrospinning to create a LLDPE fibrous matrix for efficiently removing viscous oils. It addresses the limitations of existing oil sorbents, offering high adsorption capacity, rapid adsorption, and easy retrieval using magnetic nanoparticles^9^. To overcome these challenges, the current article used of linear low-density polyethylene (LLDPE) in a sponge-like matrix form created through a thermal process in castor oil. This approach offers several advantages over traditional film-type LLDPE sorbents. Firstly, the sponge-like matrix design provides a larger surface area, enabling the sorbent to effectively absorb high-viscosity oils. Secondly, the adsorption time is significantly improved with the high sorbent capacity.

The adsorbent chosen hinges on factors like pollutant type, concentration, removal goal, and system conditions. Ad materials differ in capacity, selectivity, and cost-effectiveness for specific uses^10^. To optimize the adsorption performance, the properties of the adsorbent material play a crucial role. The surface area of the adsorbent material determines the available sites for adsorption and directly influences the adsorption capacity^11^. Materials with larger surface areas provide more active sites for the adsorption of organic pollutants, leading to higher adsorption capacities^12^. Pore distribution matters, impacting adsorbate access to the material's interior. Clear, diverse pores aid pollutant diffusion, boosting adsorption^13^. Surface chemistry played a significant role in determining the adsorption affinity and selectivity of the material^14^. The presence of functional groups on the surface can create specific interactions with the organic pollutants, promoting their adsorption^15^. Different surface chemistries can exhibit varying affinities towards specific pollutants, allowing for tailored adsorption capabilities. Surface charge is a crucial property influencing the electrostatic interactions between the adsorbent and the adsorbate^16^. Depending on the adsorbent material, the surface charge can be either positive or negative^17^. Electrostatic interactions impact adsorption, especially for charged pollutants. Adsorbent traits significantly affect adsorption. Altering them via gamma irradiation enhances capacity, selectivity, and efficiency in purifying water^18^ . The investigation of these effects is essential for the development of effective adsorbent materials for oil–water separation and environmental remediation applications.

In recent years, gamma irradiation has been recognized as a valuable method for modifying the properties of adsorbent materials, leading to enhanced adsorption capacities^19,20^. Gamma irradiation exposes materials to ionizing radiation, resulting in structural and chemical modifications^21,22^. This radiation-induced modification could affect the surface properties, pore structure, and functional groups of the adsorbent material, consequently influencing its adsorption capacity^23,24^. The use of gamma irradiation as a modification technique had offers advantages such as simplicity, scalability, and controllability^25^.

Gamma irradiation has emerged as a promising technique for modifying adsorbent materials to enhance their efficiency in the removal of organic pollutants from various mediums, including solvents and wastewater^26^. This technology offers a compelling avenue for advancing eco-friendly water pollution control methods^27^. While previous studies have highlighted the positive impact of gamma irradiation on adsorbent properties^28^, there remains a need for a more comprehensive exploration of its applications in adsorbent modification. Understanding the parameters that influence the effectiveness of gamma irradiation in optimizing adsorption was crucial for harnessing its full potential^29^. Additionally, it is essential to weigh the advantages and potential drawbacks of this technology in the context of adsorbent modification and pollutant removal. This includes considerations of cost-effectiveness, safety protocols, and the scalability of gamma irradiation from lab-scale to industrial applications.

This study proposes a novel approach to convert recycled linear low-density polyethylene (rLLDPE) into a sponge-like material (sLLDPE) suitable for oil–water separation applications. The process involves thermal treatment of the recycled LLDPE in castor oil, followed by gamma irradiation at specific doses. The thermal treatment facilitates the formation of a porous structure in the rLLDPE, while gamma irradiation induces crosslinking reactions to enhance the oil removal ability. This research aims to investigate the effects of gamma irradiation on the properties and adsorption performance of the sLLDPE material. The sLLDPE samples were characterized using scanning electron microscopy (SEM), Fourier-transform infrared spectroscopy (FTIR), and thermal stability analysis (DSC). The adsorption performances of the irradiated sLLDPE samples were evaluated against various organic solvents and oils. The novelty of this study will contribute to expanding our understanding of the effects of irradiation on polymeric materials and their application in environmental cleanup processes. The development of effective adsorbent materials for oil–water pollution removal was crucial for environmental protection, and this research aims to provide valuable insights into the potential of gamma-irradiated sLLDPE as a promising adsorbent material. In this context, state research contributes to the development of effective and sustainable adsorbent materials for oil–water pollution removal, addressing a critical environmental concern. It offers a comprehensive exploration of the sLLDPE material, its properties, and its potential applications, contributing to the broader goal of safeguarding our environment and water resources.

## Experimental

### Materials

The rLLDPE material was supplied from Urodrip Co. in Cairo, Egypt, while the castor oil was obtained from Algomhoria Chemical Co., also in Cairo, Egypt. The crude oil, gasoline oil, motor oil, pump oil, and waste oil were supplied by an Egyptian oil company situated in the Gulf of Suez, Suez Governorate. Solvents, including carbon tetrachloride, 1,2-dichloroethane, toluene, cyclohexanone, butanone, diethyl ether, n-heptanone, and methanol, in analytical grade and were purchased from El Nasr Pharmaceutical Chemical.

### Thermally treatment conversion of (rLLDPE) to sponge-like material

The thermal treatment conversion of recycled linear low-density polyethylene (rLLDPE) into a sponge-like material, referred to as sLLDPE (sponge-like linear low-density polyethylene), involved a series of sequential steps. Initially, the rLLDPE samples underwent a thorough washing process to eliminate any contaminants and impurities. Subsequently, these samples were meticulously dried to ensure complete moisture removal, after which they were precisely measured and weighed in 0.5 g portions for subsequent processing. Following this, each 0.5 g portion of the rLLDPE underwent a 30-min immersion in 50 ml of castor oil. Castor oil, acting as a plasticizer, rendered the polymer more pliable and conducive to subsequent procedures or testing. The samples were then subjected to a thermal treatment at 300 °C, with specific temperatures tailored to meet experimental requirements and achieve the desired effects on the rLLDPE. This heating process induced structural changes within the polymer, facilitating its transformation into a sponge-like material (Supplementary video 1). Subsequently, the samples were allowed to cool to room temperature, thereby promoting the solidification of the newly formed sponge-like structure. Following the structural transformation, the sLLDPE underwent sequential immersions in toluene and carbon tetrachloride, each for 60 min, and the cleaned sLLDPE samples were subsequently dried in an oven for 12 h at 55 °C. These intricate procedures collectively contributed to the successful conversion of rLLDPE into the desired sponge-like material.

### Post-irradiation process treatment of (sLLDPE)

**Figure 1:**
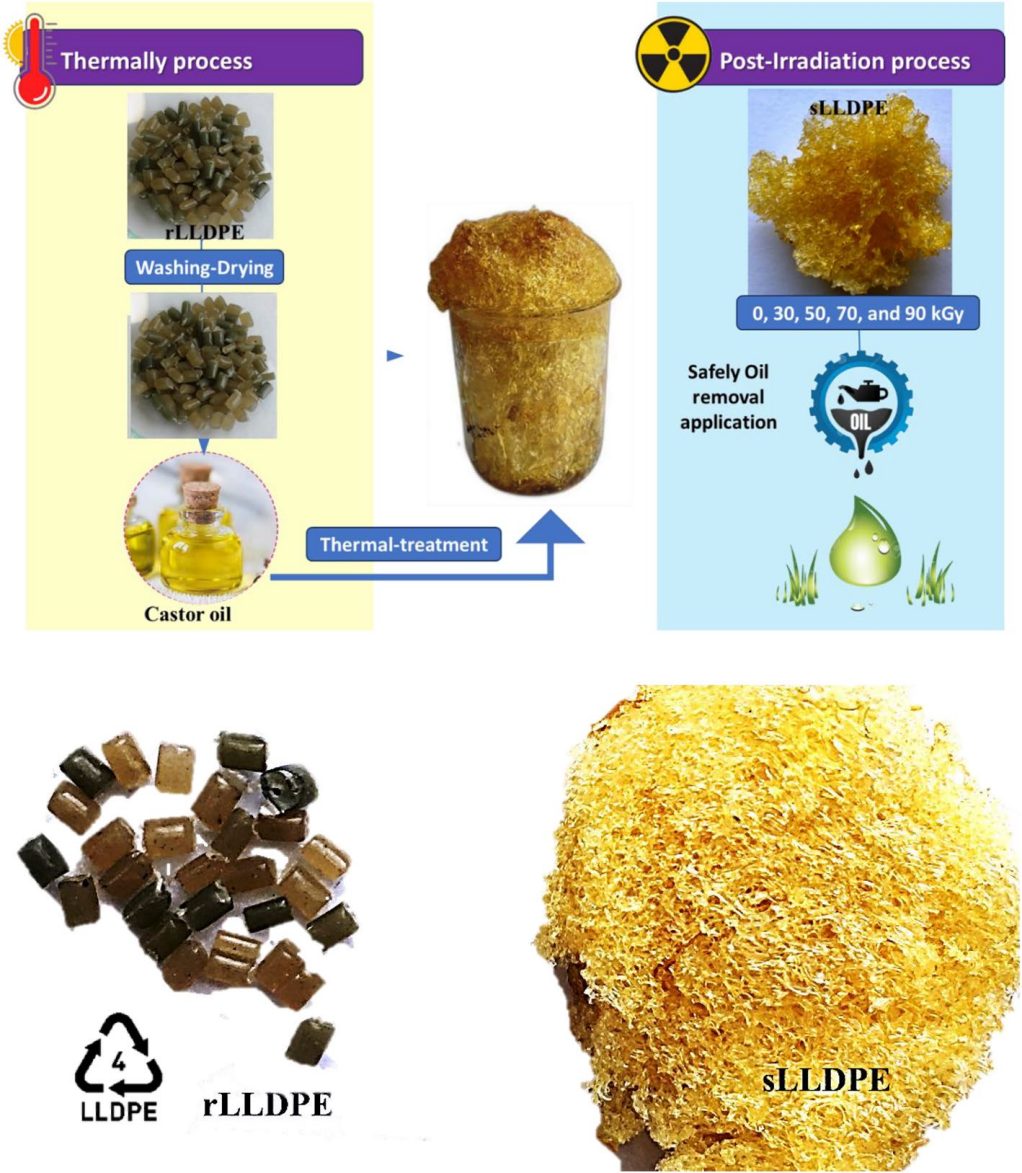
The (sLLDPE) sample, obtained after the thermal process, undergoes a post-irradiation treatment that forms a sponge-like structure for safe oil removal application.

To further modify the properties of the sLLDPE, additional post-treatment steps were performed. One such post-treatment method was the post-irradiation process. The samples of sLLDPE were exposed to ionizing radiation at doses of 30, 50, 70, and 90 kGy. The dose rate was about 0.80 kGy/h, indicating the rate at which the samples received radiation. A Co-60 γ-cell-220 source at the National Center for Radiation Research and Technology (NCRRT), Egyptian Atomic Energy Authority (EAEA), Nasr City, Cairo, Egypt, was used for the irradiation. By subjecting the sLLDPE samples to irradiation, the polymer underwent cross-linking, which affected its mechanical properties, stability, and other characteristics. The specific irradiation dose and dose rate were determined based on the desired outcomes and experimental requirements. Finally, the thermal treatment conversion process, followed by irradiation, allowed for the transformation of rLLDPE into a sponge-like material, sLLDPE, with altered properties suitable for oil removal applications. Figure 1 illustrates the post-irradiation treatment of sLLDPE to create a sponge-like structure suitable for oil removal applications. The treatment aimed to enhance the material's adsorption and retention properties, making it effective for oil spill cleanup or other oil removal tasks. Figure 1 shows the sLLDPE sample obtained after the earlier thermal process. This sLLDPE sample had already undergone heating and cooling, transforming it into a sponge-like material. The post-irradiation treatment step was performed to further modify sLLDPE and optimize its oil removal capabilities.

### Experimental setup for oil adsorption rate and uptake capacity assessment using (sLLDPE) sponge-like material

This study focused on evaluating the oil permeation capability within the pores of a sponge-like linear low-density polyethylene (sLLDPE) and its effectiveness in absorbing oil spills. The performance of the sponge sorbent was assessed through a conventional method involving the measurement of oil uptake at different time intervals. Specifically, a controlled experiment was conducted where 10% oil was introduced, and sLLDPE samples (0.1g each) were placed on the oil's surface to float. Over various time durations, ranging up to one hour, the sorbents were studied. After the designated time, the sorbents were removed, and any excess oil was allowed to drip for 30 s before their weights were measured. Equation (1) was employed to calculate the oil uptake over time, providing valuable insights into the sorbent's performance in absorbing oil under varying conditions^30^.

1 $${\mathbf{Uptake}} \;{\mathbf{capacity}} \left( {{\mathbf{g}}/{\mathbf{g}}} \right){\mathbf{Q}} = \frac{{{\mathbf{M}}_{0} - {\mathbf{M}}_{{\mathbf{s}}} }}{{{\mathbf{M}}_{{\mathbf{s}}} }}$$

In this study, the oil uptake (Q, in g/g) was calculated using Eq. (1), where M_o_ represents the post-drain sorbent mass, and M_s_ is the dry sorbent mass. The results were obtained by averaging triplicate readings to ensure accuracy and reliability. To reach equilibrium and maximize oil uptake, a one-hour contact time was established. Following this contact period, the sorbents were weighed, and their oil adsorption capacities were calculated. This experimental setup aimed to investigate the oil permeation and sorption characteristics of the sponge-like linear low-density polyethylene (sLLDPE) material. The capacity values obtained at various time intervals provided essential insights into the sorbent's efficiency in absorbing oil, which is a crucial factor in assessing its potential for oil cleanup.

### Kinetic studies of organic pollutants using sLLDPE

The kinetics of organic pollutant adsorption were investigated using pseudo-first-order and second-order kinetic models. The weight variations of the sponges immersed in organic pollutants were measured at different time intervals. To determine the first-order kinetic constant (k_1_) and second-order kinetic constant (k_2_), the experimental data obtained from the weight variations of the sponge-like material at different time intervals are fitted to the respective kinetic equations (Eqs. 2 and 3). The fitting process allows for the estimation of the kinetic constants^31^.

2 $$\ln \frac{{{\mathbf{Qm}}}}{{{\mathbf{Q}}_{{\mathbf{m}}} - {\mathbf{Q}}_{{\mathbf{t}}} }} = {\mathbf{k}}_{1} {\mathbf{t}}$$

3 $$\frac{{\mathbf{t}}}{{{\mathbf{Q}}_{{\mathbf{t}}} }} = \frac{{\mathbf{t}}}{{{\mathbf{Q}}_{{\mathbf{m}}} }} + \frac{1}{{{\mathbf{k}}_{2} {\mathbf{Q}}_{{\mathbf{m}}}^{2} }}$$

Here Q_m_ is the maximum organic pollutant adsorption capacity (g/g) and Q_t_ is the organic pollutant adsorption capacity (g/g) at time t (Min).

### Isotherm model studies of oil removal using sLLDPE

The Langmuir isotherm is a widely used model for describing the adsorption behavior of solutes onto solid surfaces ^31^. In the context of the oil adsorption experiment with the sLLDPE sponge-like material, the Langmuir isotherm can provide insights into the relationship between the quantity of oil absorbed by the sponge and the concentration of oil in the surrounding medium.

4 $${\mathbf{q}}_{{\mathbf{e}}} = \frac{{{\mathbf{q}}_{{\mathbf{m}}} {\mathbf{k}}_{{\mathbf{L}}} {\mathbf{C}}_{{\mathbf{e}}} }}{{1 + {\mathbf{k}}_{{\mathbf{L}}} {\mathbf{C}}_{{\mathbf{e}}} }}$$

where: q_e_ is the oil adsorption capacity of the sponge (g/g), q_m_ is the maximum oil adsorption capacity of the Sponge (g/g), K_L_ is the Langmuir constant related to the affinity between the oil and the Sponge. C_e_ is the oil concentration in the surrounding media (g/mL), A linear expression of the Langmuir isotherm may be represented by (5):

5 $$\frac{{{\mathbf{C}}_{{\mathbf{e}}} }}{{{\mathbf{q}}_{{\mathbf{e}}} }} = \frac{1}{{{\mathbf{q}}_{{\mathbf{m}}} {\mathbf{k}}_{{\mathbf{L}}} }} + \frac{{{\mathbf{C}}_{{\mathbf{e}}} }}{{{\mathbf{q}}_{{\mathbf{m}}} }}$$

The values of q_m_ (the maximum oil adsorption capacity of the sponge) and K_L_ (the adsorption affinity between the sponge and oil) can be determined by plotting the experimental data of $\frac{{C}_{e}}{{q}_{e}}$ against ${C}_{e}$ and they fit it into the Langmuir equation. By analyzing the Langmuir isotherm parameters, it is possible to understand the interaction between the oil and the sponge surface, the sponge's adsorption capacity, and the material's suitability for oil removal applications.

The Freundlich isotherm equation describes the adsorption of solutes onto heterogeneous surfaces and is often used to analyze experimental data. It assumes a multilayer adsorption process, where oil molecules are adsorbed onto the sponge surface at different sites with varying affinities. Equation (6) represents the Freundlich isotherm:

6 $${\mathbf{q}}_{{\mathbf{e}}} = {\mathbf{K}}_{{\mathbf{f}}} {\mathbf{C}}_{{\mathbf{e}}}^{{{\raise0.7ex\hbox{$1$} \!\mathord{\left/ {\vphantom {1 {\mathbf{n}}}}\right.\kern-0pt} \!\lower0.7ex\hbox{${\mathbf{n}}$}}}}$$

The Equation represents the linear form of the Freundlich isotherm, where K_f_ represents the Freundlich constant associated with the adsorption capacity of the sponge and "n" represents the Freundlich constant related to the intensity of adsorption.

7 $$\log {\mathbf{q}}_{{\mathbf{e}}} = \left( {\frac{1}{{\mathbf{n}}}} \right)\log {\mathbf{C}}_{{\mathbf{e}}} + \log {\mathbf{k}}_{{\mathbf{f}}}$$

The values of K_f_ (the adsorption capacity of the sponge) and n (the adsorption intensity) can be determined by plotting the experimental data against each other and fitting them to the Freundlich Equation. The Freundlich isotherm model provides insights into the nature of adsorption, including the adsorption capacity, heterogeneity of adsorption sites, and the interaction between the oil and the sponge surface. Analyzing the Freundlich isotherm parameters makes it possible to assess the suitability of the sLLDPE sponge-like material for oil adsorption applications.

### Cycling adsorption performance

The study conducted twenty cycles to evaluate the recycling and adsorption performance of the sLLDPE sponge. The process involved oil adsorption, followed by squeezing to measure oil release, with the initial capacity serving as a reference for retention. These findings offer valuable insights into the long-term effectiveness, sustainability, and potential for reuse of the sLLDPE sponge. This research contributes to the enhancement of cost-effective methods for oil cleanup and removal, making it more efficient and environmentally sustainable.

### Characterization

The chemical structure of the rLLDPE and sLLDPE was confirmed by using the Fourier transform infrared spectrophotometer (FTIR). The Vertex 70 FTIR spectrometer was equipped with HYPERION™ series microscope (Bruker Optik GmbH, Ettlingen, Germany). The thermal DSC analysis (Perkin Elmer DSC 4000) was programmed to include a heating zone between 30 and 350°C at a heating rate of 10°C/min, and Nitrogen was used as the purging gas. The surface morphology and elemental analysis were analyzed by SEM–EDX mapping (ZEISS EVO 15 microscope). The sponge surfaces were pre-coated with a thin gold layer in order to reduce charging in the SEM.

## Results and discussion

### Physiochemical properties of sLLDPE

#### FTIR analysis

**Figure 2:**
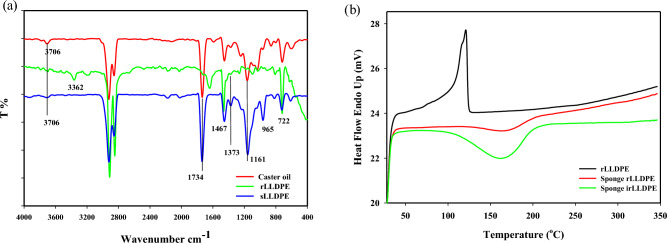
FTIR spectra (**a**) of castor oil, rLLDPE and sLLDPE, and (**b**) DSC of heating scans of rLLDPE, sponge rLLDPE and sLLDPE at 50 kGy.

The FTIR test was utilized to identify the functional groups in castor oil, rLLDPE and sLLDPE sponge, as shown in Fig. 2a. Figure 2a illustrates the outcomes FTIR analysis of castor oil. The broad peak observed at 3706 cm^−1^ corresponds to O–H bond stretching due to hydroxylated ricinoleic acid^32^. This indicated the presence of fatty acids and glycerol. The peaks around 2920–2850 cm^−1^ are due to C-H stretching vibrations from the long alkyl chains of the fatty acids and glycerol. The peaks around 1734 cm^−1^ are due to C = O stretching vibrations from the carboxylic acid and ester groups in the fatty acids and triglycerides. The peaks around 1161 cm^−1^ are due to C–O stretching vibrations from the ester groups in the triglycerides and fatty acids. Castor oil contains a high proportion of ricinoleic acid, with a cis double bond in the 12th position of the fatty acid chain. The peak around 965 cm^−1^ is characteristic of the = C–H out-of-plane bending vibration from a cis double bond. The exact position of this peak depends on the location of the double bond along the fatty acid chain. This helps identify and quantify the ricinoleic acid in castor oil^33^. The characteristic FTIR peaks of rLLDPE located at 2853 – 2954 cm^−1^ for C–H stretch in alkane groups. This is a strong and broad peak due to the overlapping C–H stretches of the –CH2– and –CH3 groups in the polymer chains. The FTIR peak at 1467 cm^−1^ is corresponding to the C–H bend in alkane groups. The peaks located at 722 cm^−1^ are attributed to C–H rock The intensity of this beak is a weak peak corresponding to the rocking motion of the C–H bonds in the aliphatic –CH2– and –CH3 groups. The FTIR peak located at 1373 cm^−1^ is attributed to C–H deformation, which indicated the branched nature of the polymer chains, characteristic of LLDPE.

The high-intensity FTIR peak at 1161 cm^−1^ is attributed to C–C stretch (polymer backbone). This peak corresponds to the asymmetric C–C stretching vibrations along the backbone of the polyethylene chains. Furthermore, the FTIR analysis of sLLDPE in Fig. 2a provides confirmation of the modification in linear polyethylene. This is evident from the presence of similar functional groups originating from castor oil within the spectrum. The FTIR spectrum of sLLDPE will show modifications compared to conventional rLLDPE due to the incorporation of castor oil. Specifically, the FTIR spectrum of sLLDPE will show the same characteristic peaks of rLLDPE related to the polyethylene chains at C–H stretches around 2853–2954 cm^−1^, C–H bends around 1462 cm^−1^ and C–C stretch around 1161 cm^−1^. However, the intensity of the 1161 cm^−1^ peak may be increased to some extent due to C–O stretching vibrations from the ester groups in castor oil. A broad O–H stretches around 3706 cm^−1^ from the hydroxyl groups in castor oil and C = O stretches around 1734 cm^−1^ from the ester groups and fatty acids in castor oil. A new peak around 960 cm^−1^ corresponds to the = C–H out-of-plane bend from the cis double bond in ricinoleic acid, the main fatty acid in castor oil. So, in summary, the FTIR spectrum of sLLDPE produced from castor oil will show both the characteristic peaks of rLLDPE from the polyethylene chains and additional peaks related to the functional groups and double bonds present in castor oil. These modifications confirm that castor oil has been successfully incorporated into the sLLDPE.

#### DSC analysis

The Fig. 2b represents the thermal DSC analysis performed on the rLLDPE as received and two sLLDPE samples at two irradiation doses (0 and 50 kGy). It represents the melting point (Tm), enthalpy change (ΔH), and area under the curve (mJ) for each sample. For rLLDPE blank as received, exhibits a melting point (Tm) at 121.03°C^34^, with an enthalpy change (ΔH) of 129.95 J/g. The area under the curve, representing the total heat absorbed during the melting process, is 389.8 mJ.

For the sLLDPE sample irradiated at 0 kGy, a new exothermic peak is observed instead of the melting peak (Tm) in rLLDPE. This new peak, denoted as the curing point (Tc) similar to the curing point (Tc) of polyurethane sponge^35^, has a value of 168.28 °C. The enthalpy change (ΔH) associated with this transition is negative (− 37.14 J/g), indicating heat released during curing. The area under the peak is − 111.42 mJ, representing the heat released during the curing transition. In the case of the sLLDPE sample irradiated at 50 kGy, a similar exothermic peak (Tc) is observed at 163.3 °C. The enthalpy change (ΔH) associated with this transition is increased and becomes − 167.92 J/g, indicating a larger amount of heat released during the curing process. The area under the curve is -503.37 mJ, representing the heat released during the curing transition. The thermal DSC analysis reveals changes in the melting behavior of the rLLDPE sample compared to the sLLDPE samples.

Exothermic peaks in the sLLDPE samples indicate a curing process occurring at temperatures ranging from 163.3 to 168.28 °C. The negative enthalpy changes values (ΔH) and the heat released during the curing process suggest the exothermic nature of the cross-linking reactions^36^. This behavior is similar to what is observed in the DSC thermograms of polyurethane sponge materials. Moreover, the increase in enthalpy change (ΔH) after the irradiation process (50 kGy) compared to 0 kGy suggested changes in the crystallinity and thermal properties of the sLLDPE material due to irradiation^37^. Gulati et al.^38^ Studied the effect of gamma irradiation on the properties of linear low-density polyethylene (LLDPE). Irradiation with 75 kGy of gamma rays improved the tensile strength and thermal stability compared to the unirradiated samples and those irradiated with 150 kGy. The presence of plasticizer compounds in the recycled LLDPE can undergo radiolytic reactions during irradiation^39^. These reactions can form radical groups, which easily attach to the polymer chains and induce internal changes within the material. The resulting modifications in crystalline structure and thermal behavior are reflected in the observed changes in melting point (Tm), curing point (Tc), and enthalpy change (ΔH) in the DSC analysis^40^. The specific types of radical groups formed during radiolytic reactions can vary depending on the nature of the plasticizer compounds and irradiation conditions^41^ . These radical groups, such as alkyl, aryl, and acyl radicals, are highly reactive and could affect the polymer's structure and properties by branching or crosslinking the polymer chains^42^.

On the other hand, the increase in enthalpy changes after the irradiation process (50 kGy) compared to (0 kGy) suggested changes in the crystallinity and thermal properties of the material due to irradiation. The irradiation process can indeed affect the enthalpy change (ΔH) of LLDPE samples, especially in the case of recycled plastic materials that may contain plasticizer compounds. During irradiation, the plasticizer compounds in the recycled LLDPE can undergo radiolytic reactions. These reactions can lead to forming radical groups, which can easily attach to the main chains of the polymer. The attachment of these radical groups to the polymer chains can induce internal changes within the material. The crystalline structure of the polymer may be affected, leading to alterations in the melting point (Tm) and enthalpy change (ΔH) observed in the DSC analysis. The increased enthalpy change indicated changes in the energy required for the thermal transition. This can be attributed to modifications in the crystallinity and thermal behavior induced by the radiolytic reactions and the attachment of radical groups^43^. Examples of radical groups that can be formed during radiolytic reactions in the presence of plasticizer compounds include alkyl radicals (such as methyl, ethyl, or butyl radicals), aryl radicals (such as phenyl or benzyl radicals), and acyl radicals (such as acetyl or benzoyl radicals). These radical groups are highly reactive and can easily attach themselves to the polymer chains, leading to modifications in the structure and properties of the material. The attachment of alkyl radicals, for instance, can result in branching or crosslinking of the polymer chains, affecting the crystallinity and thermal behavior of the material. It's important to note that the specific types of radical groups formed during radiolytic reactions can vary depending on the nature of the plasticizer compounds and the irradiation conditions. The resulting radical groups can contribute to internal changes within the polymer matrix, influencing its properties, including melting point (Tm), curing point (Tc) and enthalpy change (ΔH).

#### SEM analysis

**Figure 3:**
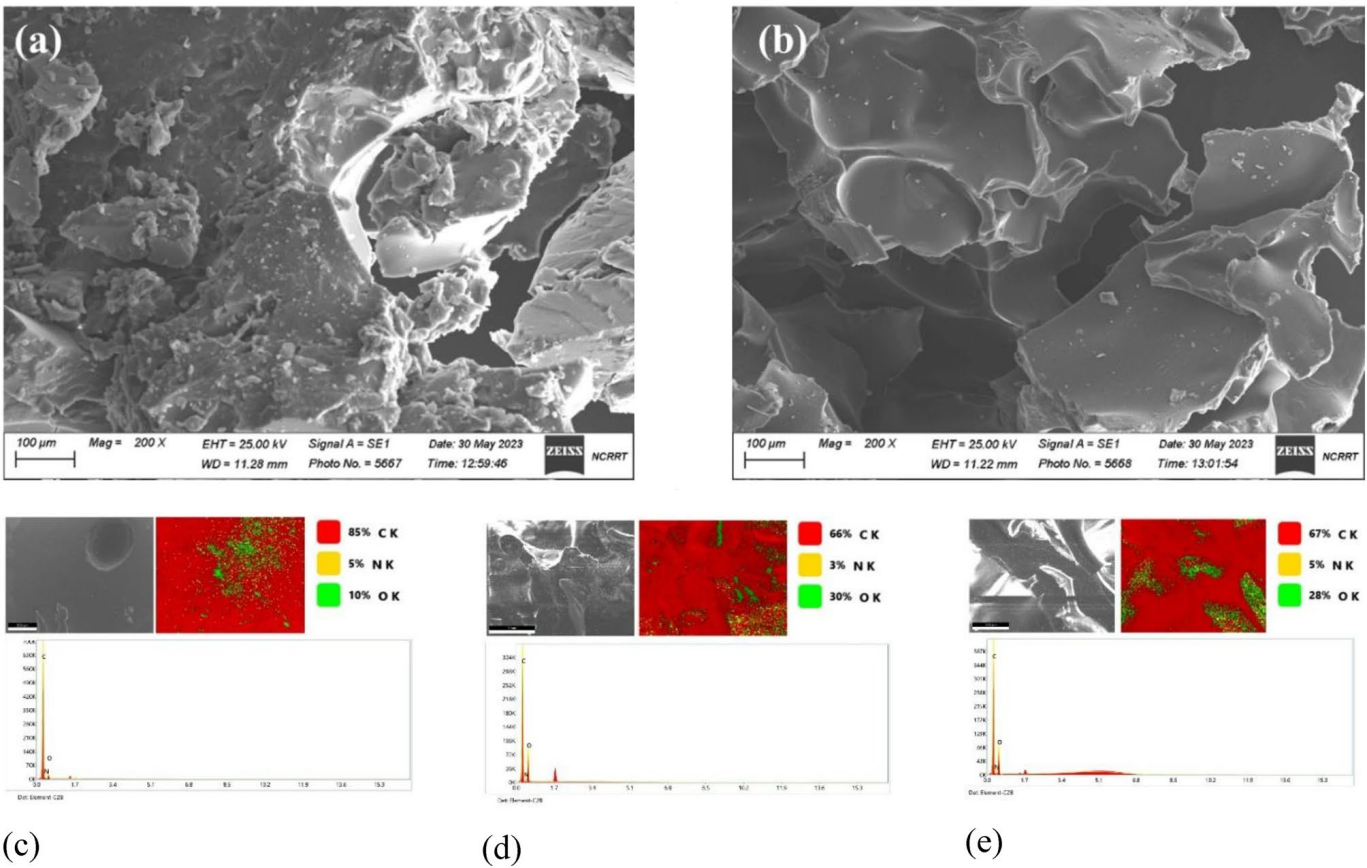
SEM image of sLLDPE irradiated at (**a**) 0 kGy, (**b**) 50 kGy and EDX mapping analysis of (**c**) rLLDPE, and (**d**, **e**) sLLDPE irradiated at 0 and 50 kGy, respectively.

The Fig. 3a,b show the SEM image of sLLDPE (irradiated at 0 and 50 kGy). The SEM images of sLLDPE samples irradiated at 0 kGy and 50 kGy revealed a large, random-shaped porous structure. This porous morphology is visually apparent in the SEM images, indicating changes in the surface morphology of the sLLDPE samples due to the irradiation process. The random-shaped pores observed in the SEM images suggest cross-linking reactions and forming a three-dimensional network structure within the sLLDPE material. The irradiation process induces the formation of radicals, which can react and bond with the polymer chains, creating new cross-links. These cross-links contribute to developing the porous structure by introducing void spaces or gaps within the polymer matrix. The presence of such pores in the sLLDPE material can have significant implications for its properties and applications. The porous structure can enhance the material's surface area, allowing for increased interactions with other substances or improved adsorption capabilities. It can also affect mechanical properties such as tensile strength, as the presence of pores can introduce structural weaknesses. The porous microstructure created after surface modification and gamma irradiation is likely responsible for the increased surface area and more functional groups observed in these samples. The pores allow access of reactants during surface modification and space for more functional groups to form. The porous structures become more prominent and developed after gamma irradiation at 50 kGy compared to the 0 kGy sample. This suggested that gamma irradiation further induces the formation of pores within the LLDPE material, possibly by breaking bonds and inducing chain scission. Figure 3c,d,e showed EDX mapping analysis of rLLDPE, sLLDPE (0 kGy) and sLLDPE (50 kGy).

Raw LLDPE has the highest carbon content at 85% and the lowest oxygen at 10%. This is expected as LLDPE is a hydrocarbon polymer. After surface modification in castor oil, the oxygen content increases to 30% for untreated LLDPE (0 kGy) while carbon decreases to 66%. This indicated that oxygen-containing functional groups are introduced onto the LLDPE surface during surface modification. The oxygen content decreases slightly to 28% for LLDPE irradiated at 50 kGy, while nitrogen increases from 3 to 5%. This suggested that gamma irradiation introduces additional functional groups onto the surface. The oxygen and nitrogen content increase after surface modification and gamma irradiation correlates with the FTIR results, which detected new functional groups. The functional groups contain oxygen and possibly nitrogen, accounting for the changes seen in EDX mapping. These functional groups are expected to improve the adsorption capacity of LLDPE for pollutant removal from water.

### Effect of gamma irradiation dose on Adsorption Capacity of organic pollutant by (sLLDPE)

**Figure 4:**
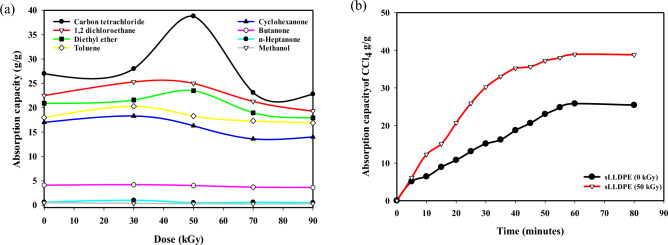
The effect of gamma irradiation dose on the adsorption capacity of organic pollutants by (sLLDPE) (**a**). The Adsorption rate of carbon tetrachloride by sLLDPE Irradiated at Different radiation doses (0 and 50 kGy) (**b**).

The sLLDPE evaluated the effect of gamma irradiation on sLLDPE for its adsorption performance against 8 types of organic solvents, and the results in Fig. 4a and Table 1 indicate the maximum amount of each solvent that can be adsorbed by the sLLDPE irradiated at a dose of (0, 30, 50, 70 and 90 kGy). For example, at an irradiation dose of 0 kGy, the sLLDPE material can adsorb a maximum of 27 g/g of carbon tetrachloride, 22.5 g/g of 1,2 dichloroethane, 20.91 g/g of diethyl ether, 18 g of toluene, 17 g/g of cyclohexanone, 4.1 g/g of butanone, 0.6 g/g of n-heptanone, and 0.5 g/g of methanol. At an irradiation dose of 50 kGy, the maximum amount of each solvent that the sLLDPE material can adsorb is as follows: Carbon tetrachloride: 38.8 g/g, 1,2 dichloroethane: 25 g/g, diethyl ether: 23.5 Toluene: 18.3 g/g, Cyclohexanone: 16.3 g/g, Butanone: 4.02 g/g, n-Heptanone: 0.5 g/g and Methanol: 0.3 g/g. At an irradiation dose of 90 kGy, the maximum amount of each solvent that the sLLDPE material can adsorb is as follows: Carbon tetrachloride: 22.8 g/g, 1,2 dichloroethane: 19.3 g/g, diethyl ether: 17.94 g/g, Toluene: 16.9 g/g, Cyclohexanone: 14 g/g, Butanone: 3.62 g/g, n-Heptanone: 0.51 g/g and Methanol: 0.3 g/g. Comparing the maximum sorption capacities of the sLLDPE material at different irradiation doses, we can observe the following trends: Carbon tetrachloride: The sorption capacity increased from 27 g/g at 0 kGy to 38.8 g/g at 50 kGy, and then decreased to 22.8 g/g at 90 kGy. 1,2 dichloroethane: The sorption capacity slightly increases from 22.5 g/g at 0 kGy to 25 g/g at 50 kGy, then decreases to 19.3 g/g at 90 kGy. Diethyl ether: The sorption capacity increased from 20.91 g/g at 0 kGy to 23.5 g/g at 50 kGy, then decreased to 17.94 g/g at 90 kGy. Toluene: The sorption capacity increases from 18 g/g at 0 kGy to 18.3 g/g at 50 kGy, and then decreases to 16.9 g/g at 90 kGy. Cyclohexanone: The sorption capacity decreases from 17 g/g at 0 kGy to 16.3 g/g at 50 kGy, and further decreases to 14 g/g at 90 kGy. Butanone: The sorption capacity decreases from 4.1 g/g at 0 kGy to 4.02 g/g at 50 kGy, decreasing to 3.62 g/g at 90 kGy. The sorption capacity remains relatively constant of n-Heptanone and Methanol ⁓ 0.6 g/g and 0.5 g/g across all irradiation doses (0, 30, 50, and 90 kGy). The sorption capacities of the sLLDPE material generally increase as radiation doses increase. By increasing the radiation dose over 50 kGy, the absorption capacity decreases; this could be due to a crosslinked reaction induced by gamma irradiation^44^. Gamma irradiation can cause the formation of crosslinks between polymer chains, resulting in a more rigid and less porous structure^45^. This reduced porosity can lead to a decrease in the material's ability to absorb and retain.

**Table 1:** show the effect of gamma irradiation on the adsorptive capacity of sLLDPE against different organic solvents.

Dose (kGy)	Carbon Tetrachloride (g/g)	1,2 Dichloroethane (g/g)	Diethyl Ether (g/g)	Toluene (g/g)	Cyclohexanone (g/g)	Butanone (g/g)	n-Heptanone (g/g)	Methanol (g/g)
0	27.0	22.5	20.91	18.0	17.0	4.10	0.60	0.50
50	38.8	25.0	23.50	18.3	16.3	4.02	0.50	0.30
90	22.8	19.3	17.94	16.9	14.0	3.62	0.51	0.30

Based on the classification of solvents and their corresponding adsorption capacities by sLLDPE (50 kGy). These results indicate the adsorption capacity of sLLDPE (50 kGy) for each solvent according to its polarity classification. It can be observed that the adsorption capacity varies among different solvent classes, with higher adsorption capacities observed for non-polar solvents such as carbon tetrachloride, moderately polar solvents such as toluene and cyclohexanone, and polar aprotic solvent 1,2 dichloroethane through (physisorption) process. On the other hand, polar protic solvents, such as methanol, and polar solvents, such as n-heptanone, exhibit relatively lower adsorption capacities. This trend in solvent adsorption by sLLDPE can be explained by the relative polarity of the solvents. More polar solvents are absorbed less than non-polar or slightly polar solvents. The sLLDPE material is composed of non-polar polyethylene chains. These polyethylene chains are hydrophobic and tend to exclude polar solvents. They can, however, absorb non-polar and slightly polar solvents into the spaces between the chains. Among the solvents listed, carbon tetrachloride and 1,2-dichloroethane are the least polar. They have the highest adsorption amounts of 38.8 g/g and 25 g/g, respectively. These chlorinated hydrocarbons can easily fit between the polyethylene chains. As the polarity of the solvents increases, their adsorption by sLLDPE decreases. Toluene, diethyl ether, and cyclohexanone, which are slightly polar, show lower adsorption of 23.5 g/g, 18.3 g/g and 16.3 g/g. Ketones like butanone and n-heptanone, which are more polar, are absorbed even less. Methanol, as a strongly polar protic solvent, showed the lowest adsorption of just 0.3 g/g. The hydroxyl group and hydrogen bonding ability of methanol make it incompatible with the non-polar polyethylene chains. In summary, the trend in adsorption can be attributed to the solvents' relative polarity and the sLLDPE material's hydrophobic nature.

The Fig. 4b displays carbon tetrachloride adsorption by sLLDPE sponge at 0 and 50 kGy doses, over time (mins). Both samples' adsorption capacity rises over time, suggesting more molecules adhere as contact increases. Notably, irradiated (50 kGy) samples outperform unirradiated across most times. This irradiation boost is possibly due to radiolytic reactions with plasticizers, forming attachment groups. These groups create extra adsorption sites on the sponge, elevating its capacity for CCl_4_. Affinity between introduced groups and carbon tetrachloride likely spurs this. Note that radical groups differ with plasticizer types and irradiation conditions, altering the sponge's surface. This improved adsorption is likely due to irradiation. Attached groups, such as methyl radicals, aid enhancement.

### Evaluate the oil adsorption capacity by irradiated sLLDPE

**Figure 5:**
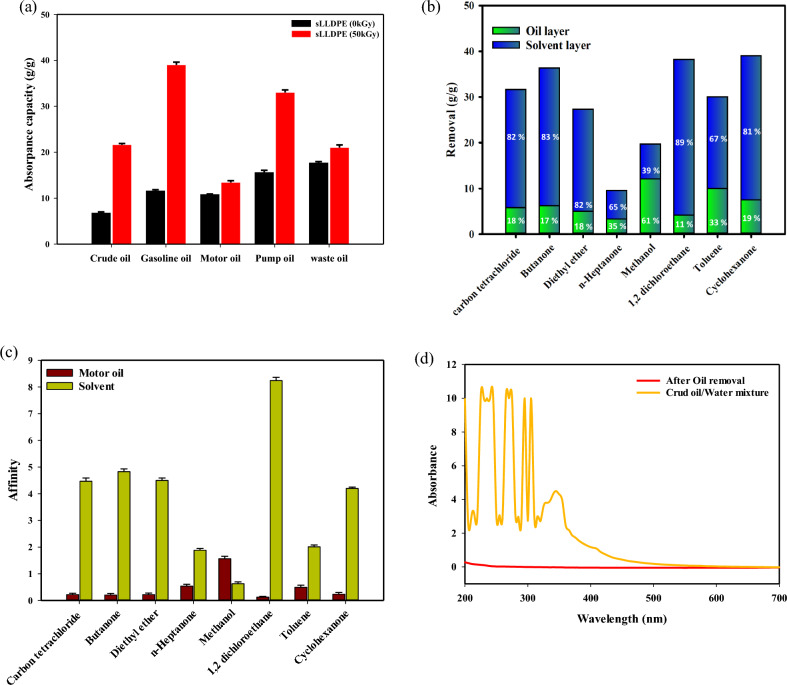
The adsorption capacity of various oils with sponge sLLDPE irradiated at doses of 0 and 50 kGy (**a**), removing various organic contaminants from oil-based systems (**b**). and the affinity values provide insights into the relative preference of the sLLDPE sponge irradiated at 50 kGy towards the motor oil compared to the solvent components (**c**) and the UV spectrophotometer of the crude oil/water emulsion (**d**).

The Fig. 5a presents the oil adsorption capacities of sLLDPE (0 kGy) and sLLDPE (50 kGy) sponges for different types of oils. The results indicate that the adsorption capacity varies depending on the type of oil. It can be observed that the irradiated sLLDPE (50 kGy) sponge generally exhibits higher adsorption capacities for all types of oils compared to the unirradiated sLLDPE (0 kGy) sponge. This suggests that the irradiation process at a dose of 50 kGy enhances the sponge's adsorption properties, making it more effective in capturing and retaining oils. Gamma irradiation causes the formation of free radicals within the polymer matrix. These free radicals can react with the surrounding molecules, introducing additional functional groups or modifications in the polymer chains. The adsorption capacity of the sLLDPE (50 kGy) sponge is approximately 216.2% higher compared to the sLLDPE (0 kGy) sponge. In the case of sLLDPE, the irradiation process can result in the attachment of new groups, such as methyl groups, through radiolytic reactions involving plasticizer compounds. The attachment of these groups can enhance the sponge's affinity for oils and improve its adsorption capacity. Methyl groups, for example, are known to be hydrophobic and can provide increased oil-attracting properties. This can facilitate the adsorption and retention of oil molecules within the sponge structure.

Additionally, the irradiation process can induce crosslinking or branching of the polymer chains, leading to an increased porosity and surface area of the sponge. This increased porosity allows for greater oil accessibility and provides more sites for oil adsorption, further enhancing the adsorption capacity of the irradiated sLLDPE sponge. It is important to note that the adsorption capacity can vary depending on the type of oil. Gasoline oil: The adsorption capacity of the sLLDPE (50 kGy) sponge is approximately 235.3% higher compared to the sLLDPE (0 kGy) sponge. Motor oil: The adsorption capacity of the sLLDPE (50 kGy) sponge is approximately 24.1% higher compared to the sLLDPE (0 kGy) sponge. Pump oil: The adsorption capacity of the sLLDPE (50 kGy) sponge is approximately 111.5% higher compared to the sLLDPE (0 kGy) sponge. Waste oil: The adsorption capacity of the sLLDPE (50 kGy) sponge is approximately 18.6% higher compared to the sLLDPE (0 kGy) sponge. Different oils have different chemical compositions and properties, such as viscosity, density, and surface tension, which can influence their interaction with the sponge material. Motor oil and waste oil have less adsorption quantity because they contain more polar additives that are less compatible with the non-polar sponge material, even after irradiation treatment. Hence, the variations in adsorption capacities observed for different types of oils in Fig. 5a can be attributed to the specific characteristics of each oil. This suggested that irradiation enhances the effectiveness of the sponge in absorbing and removing oils from the environment. Removing various organic contaminants from oil-based systems shown in Fig. 5b. The sLLDPE irradiated at 50 kGy demonstrates effective removal of various organic contaminants from oil-based systems, as shown by the adsorption capacities for different solvents. From the Fig. 5b, we can observe that the percentage of oil varies for different solvents. Solvents like carbon tetrachloride and butanone have higher percentages of solvent (81.71% and 82.85%, respectively) and lower percentages of oil (18.29% and 17.15%, respectively).

On the other hand, solvents like methanol have higher percentages of oil 61.45% and lower percentages of solvent 38.55%. Figure 5c shows a higher affinity value indicating a stronger interaction or adsorption capacity for the oil component in polar solvent. In this case, solvents like methanol exhibit higher affinity values of 1.595, indicating a stronger affinity towards the oil component. On the other hand, oil affinity in solvents like 1,2 dichloroethane and carbon tetrachloride have lower affinity values (0.121 and 0.219, respectively), suggesting a relatively weaker interaction with the oil component. This could be due to oil being more viscose than solvent. The affinity values provide a relative measure of the sponge's interaction with and preference for extracting different solvents from the motor oil. Higher affinities correlate with better solvent extraction performance. The UV–visible spectroscopy analysis presented in Fig. 5d provides valuable insights into the performance of the sLLDPE sponge irradiated at 50 kGy as an oil adsorbent for removing crude oil from water. The spectrum of the crude oil solution (100 mg L^−1^) in a water/surfactant mixture exhibits peaks in the UV range (between 200 and 700 nm), indicating the presence of certain components or functional groups in the crude oil. However, after applying the sLLDPE sponge and subsequent removal of crude oil from the water, the peaks observed in the UV–visible spectrum disappeared. This disappearance of peaks signifies the efficient removal of crude oil from the water/surfactant mixture using the sLLDPE sponge. The sLLDPE sponge effectively adsorbs the crude oil, thereby leading to the elimination of its UV-absorbing components. The complete removal of crude oil demonstrated by the disappearance of UV peaks confirms the high efficiency of the adsorption process. It indicated that the sLLDPE sponge has a strong affinity for crude oil and can effectively capture and retain it from water, even at trace levels. This UV–visible spectroscopy analysis provides experimental evidence supporting the sLLDPE sponge's effectiveness as an oil adsorbent, highlighting its potential for various oil spill cleanup applications and environmental remediation efforts.

### Oil/water separation performance of sLLDPE (50 kGy)

**Figure 6:**
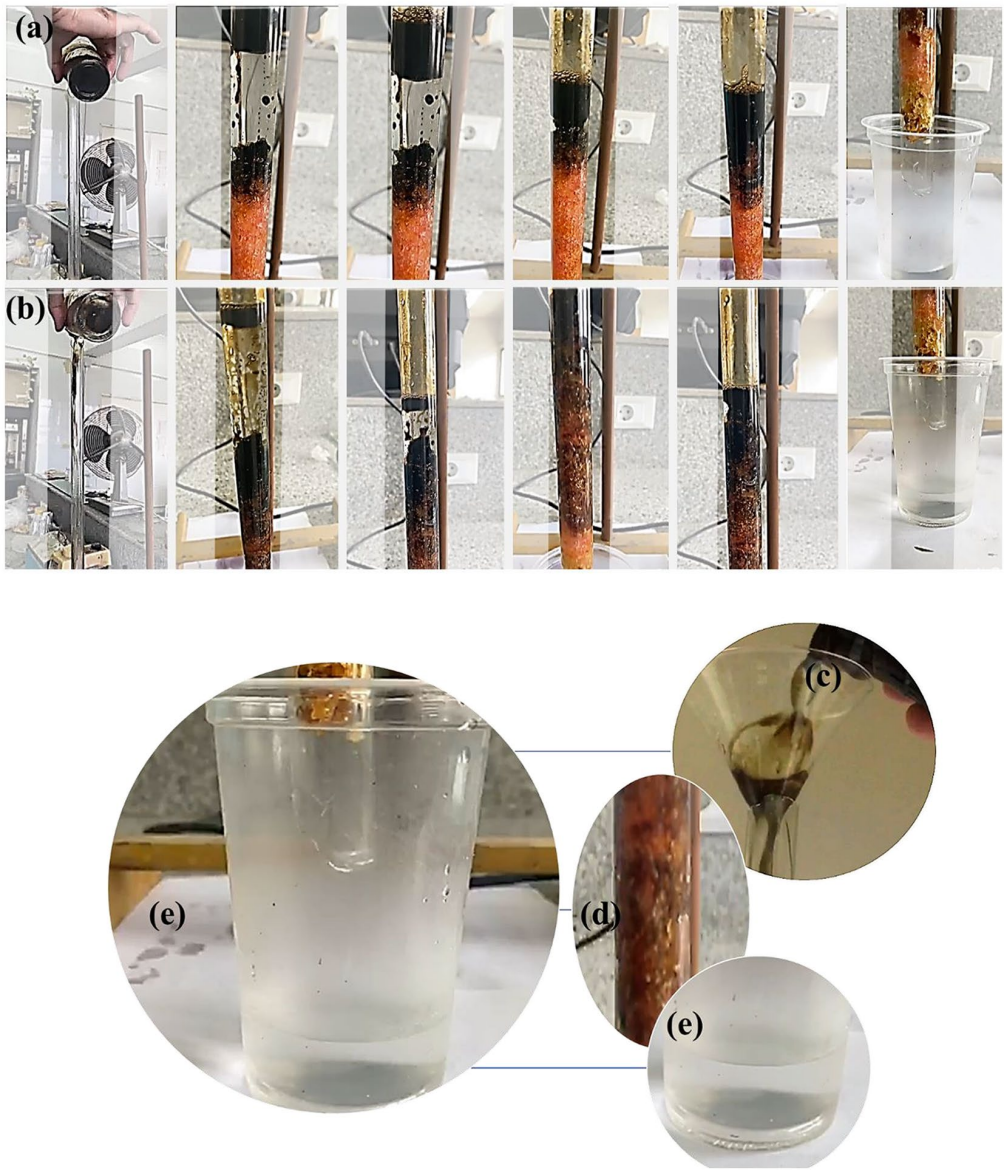
Demonstrates the video snapshots of crude Oil/water a quick separation (**a**) and (**b**) continuous quick separation for a second time using the sLLDPE sponge (50 kGy) in the column, (**c**) a mixture of crude oil and water, (**d**) the column contains sLLDPE sponge (50 kGy) and (**e**) the collected water (100% pure) from the column separation process.

The Fig. 6a,b demonstrates the oil–water separation performance of sLLDPE (50 kGy) sponge material. In Fig. 6a, the contaminated water containing crude oil passed through the sLLDPE (50 kGy) sponge under gravity, quickly separating oil from water. The sponge effectively retained the oil, while the clear water was collected in a cup. This process was repeated for a second time in Fig. 6b, with the oil being separated and the water collected again. The results indicated that the sLLDPE (50 kGy) sponge exhibited an ultrafast oil–water separation rate, achieving a separation efficiency of approximately 100% (Supplementary Video 2 and Video 3).

The continuous separation of oil–water mixtures was crucial for practical applications, and the superhydrophobic sLLDPE (50 kGy) sponge material was evaluated for its performance in Fig. 6b. During the separation process, the sponge material completely retained the oil. In comparison, the water rapidly passed through the pores of the sLLDPE (50 kGy) sponge, flowing into the outlet and collecting in the cup. This demonstrates the efficient and effective separation capability of the sLLDPE (50 kGy) sponge, highlighting its potential for real-world oil–water separation applications.

A self-made column for oil–water separation was demonstrated in Fig. 6c,d,e. This demonstrates the practical application of the sLLDPE (50 kGy) sponge material in the self-made column for efficient oil–water separation. The process allows for the separation of oil and water, with the water being collected for further use or disposal. The sLLDPE sponge effectively acts as a filter, trapping the oil while allowing the water to pass through, successfully separating the two components. In Fig. 6c, a 100 mL mixture of crude oil and water in a volume ratio 1:1 was prepared. In Fig. 6, a suitable amount of sLLDPE (50 kGy) sponge material was placed in a glass column. The mixture of oil and water was then slowly poured into the column glass, allowing the separation process to occur. In Fig. 6e, the filtered liquid, consisting of separated water, was collected in a cup. The results in Fig. 6 illustrate the superior oil–water separation performance of the sLLDPE (50 kGy) sponge material, indicating its suitability for rapid and efficient removal of oil contaminants from water (Supplementary Video 4).

### The adsorption kinetics study

**Figure 7:**
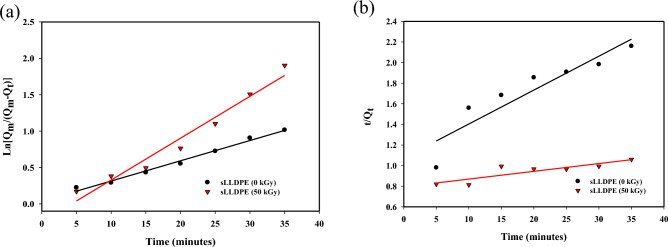
Absorption kinetic with pseudo-first-order adsorption linear fitting (**a**) and Pseudo second-order adsorption linear fitting (**b**) of the sLLDPE at radiation dose of 0 kGy and 50 kGy.

The kinetics of CCl4 adsorption on irradiated sponge-like linear low-density polyethylene (sLLDPE) samples subjected to 0 kGy and 50 kGy irradiation doses were investigated using pseudo-first-order and pseudo-second-order models. The results, as depicted in Figs. 7a,b, revealed a rapid adsorption of CCl4, reaching equilibrium within 60 min for both irradiation doses. When comparing the goodness of fit represented by R^2^ values for both models, the pseudo-first-order model exhibited higher R^2^ values (0.9885 for 0 kGy and 0.9657 for 50 kGy), indicating its superior performance in describing the adsorption process. This suggests that physical adsorption predominantly governs the adsorption of CCl4 and other solvents, which aligns with the data presented in Fig. 7. Furthermore, the comparison of rate constants, K1 and K2, revealed interesting insights. Higher K1 values indicate faster adsorption, and in this case, both the irradiated samples (0 kGy and 50 kGy) exhibited higher K1 values. This suggests that irradiation accelerates the adsorption process while retaining the pseudo-first-order kinetics. On the other hand, the lower K2 value observed for the 50 kGy irradiated sample implies enhanced hydrophobicity in sLLDPE. This increased hydrophobicity is attributed to the radiation-induced methyl groups, which attract nonpolar solutes like CCl4. Moreover, the crosslinking and branching effects induced by radiation on the polyethylene chains create a more hydrophobic, larger-pore structure in the sLLDPE, favoring the adsorption of nonpolar CCl4. These findings shed light on the intricate interplay between radiation, surface characteristics, and solute adsorption, contributing to a comprehensive understanding of the adsorption mechanisms in sLLDPE.

### The isothermal adsorption studies

**Figure 8:**
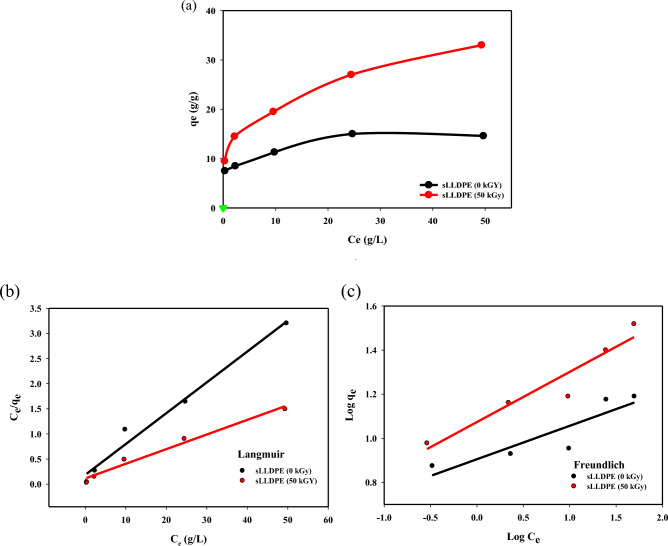
Langmuir and Freundlich adsorption isotherm models for the adsorption of gasoline oil by sponge rLLDPE and sLLDPE.

Adsorption isotherms in Fig. 8 illustrate the relationship between how much adsorbate (Gasoline oil) is removed from the liquid phase and the amount of adsorbent (sLLDPE sponge) at a given temperature. They provide a mathematical model of the adsorption process that can be used to predict and optimize adsorption system design. Figure 8a shows the experimental data for the adsorption of Gasoline oil in different quantities on sLLDPE samples irradiated at 0 kGy and 50 kGy. The parameters q_max_ (maximum adsorption capacity) and Ce (equilibrium concentration) are measured for each sample. q_max_ values almost reach the equilibrium at a certain (Ce), indicating the maximum amount of oil that can be adsorbed per unit mass of the sLLDPE material. For the sLLDPE (50 kGy) sample, the q_max_ values do not appear to reach a true equilibrium even at the highest concentration of 33 g/L.

In contrast, the q_max_ values for the 0 kGy sample seem to level off and approach equilibrium above around 15 g/L. In addition, the q_max_ does not reach the equilibrium at different (Ce) values ranging from 0.29 g/L to 49.32 g/L, showing the maximum adsorption capacity of gasoline oil on the irradiated sLLDPE material than unirradiated sLLDPE material. This could be due to the irradiation process leading to the increased active site. These active sites or surface modifications can enhance the interaction between the material and oil, resulting in a higher adsorption capacity. However, further analysis and investigation are necessary to fully understand the mechanisms and underlying factors contributing to the increased adsorption capacity of the sLLDPE (50 kGy) sample for oil.

Equilibrium parameters derived from isotherms provide insights into the adsorption mechanism, surface properties, and affinity of the adsorbent for the adsorbate shown in Fig. 8b,c. Common isotherm models like Langmuir and Freundlich can be fitted to experimental equilibrium data to determine which model best describes the adsorption system. The Langmuir model assumes monolayer adsorption onto a homogeneous surface with equivalent sites. The Freundlich model assumes heterogeneous adsorption onto a non-uniform surface. Determining which isotherm model provides the best fit can help elucidate the nature of the adsorbent-adsorbate interaction. In Fig. 8, Langmuir and Freundlich isotherms were used to analyze the equilibrium adsorption data for gasoline oil on the sLLDPE sponge. Comparing the fits of the two models can reveal whether the adsorption is better described by homogeneous monolayer adsorption (Langmuir) or heterogeneous multilayer adsorption (Freundlich)^46^. The findings presented in Table 2 indicate that the equilibrium adsorption capacity of sLLDPE samples irradiated at different doses (0 kGy and 50 kGy) was analyzed using Langmuir and Freundlich isotherm models. The results in Table 2 demonstrate that the Langmuir isotherm model better fits the equilibrium data, as indicated by higher *R*^*2*^ values than the Freundlich model. The maximum adsorption capacity (q_max_) increases with an irradiation dose of up to 50 kGy, suggesting an optimal irradiation dose for enhancing adsorption performance. Based on the higher R^2^ values for the Langmuir model, it can be concluded that the adsorption process on the sLLDPE sponge samples follows a monolayer adsorption mechanism and that the surface is relatively homogeneous. The Langmuir model assumptions align with the experimental data, indicating that the adsorption capacity reaches a maximum value at a certain adsorbate concentration in a sample of (0 kGy) as shown in Fig. 8a. Consequently, irradiation increases the maximum adsorption capacity (higher q_max_), likely by creating a more open pore structure and introducing an active site that enhances interactions with gasoline oil molecules. Irradiation treatment can optimize this adsorption process by maximizing the q_max_ value.

**Table 2:** Comparisons between Freundlich and Langmuir adsorption isotherm constants for sLLDPE at different radiation doses.

Isotherm models	sLLDPE (0 kGy)	sLLDPE (50 kGy)
Langmuir		
q_max_ (g/g)	16.39	34.36
K_L_	0.329	0.253
R^2^	0.9806	0.9802
Freundlich		
N	6.51	4.54
R^2^	0.799	0.911
K_f_	8.06	12.73

### Reusability studies

**Figure 9:**
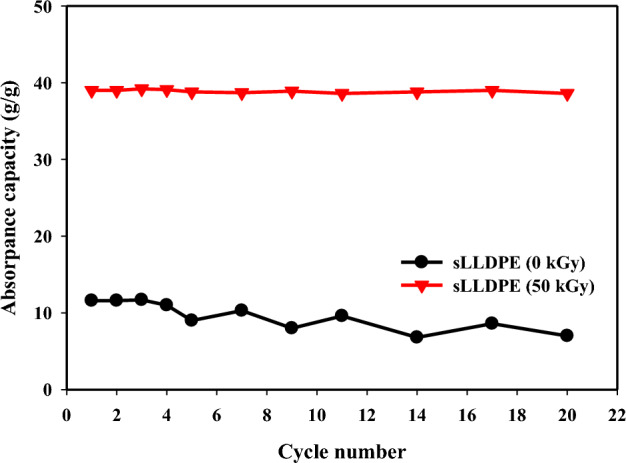
Reusability of gasoline oil absorption by sLLDPE irradiated by (0 kGy and 50 kGy) dose.

Reusability refers to the ability of a material or adsorbent to maintain its performance and effectiveness over multiple cycles of use. In the context of oil–water separation, reusability refers to the capacity of the adsorbent material, in this case, the sLLDPE sponge, to be used repeatedly to remove oil contaminants without significant loss of its adsorption properties. In the presented study, the reusability of the sLLDPE samples irradiated at 0 kGy and 50 kGy was evaluated by measuring the removal efficiency of gasoline oil over multiple recycling cycles. The removal efficiency values obtained in each cycle were compared to assess the ability of the sLLDPE material to maintain its oil adsorption capacity. Figure 9 displays gasoline oil removal by sLLDPE samples over 20 cycles. The 50 kGy irradiated sample consistently surpasses the unirradiated one in removal efficiency across cycles. The sLLDPE (50 kGy) experiences a mere 0.76% reduction in absorption capacity, while the sLLDPE (0 kGy) sees a significant 23.07% decline. After completing 20 cycles, both the sLLDPE 50 kGy and 0 kGy exhibit absorption efficiency decreases of 1.27% and 40.17%, respectively. The irradiated sLLDPE maintains high removal efficiency, indicated lasting adsorption capacity. Gamma irradiation enhances material durability and performance, suitable for repeated oil–water separation. Reusable sLLDPE holds practical value, reducing waste and costs by sustained capacity, minimizing replacements^47^.

### Comparative study

Table 3 compares various materials' absorption capacities for removing oils and organic solvents. The absorption capacities listed demonstrate the effectiveness of these materials in adsorbing and removing different types of oils and organic solvents. The sLLDPE sponge, as studied in the current research, exhibits an absorption capacity of 38.8–39 g/g for carbon tetrachloride and gasoline oil. Evaluating the factors, such as absorption capacity, availability, cost, regenerability, and mechanical properties, a comprehensive comparison can be made to determine the most suitable material for a specific oil or solvent and application. When comparing the 14 examples listed in Table 3, it is important to consider the factors mentioned earlier. sLLDPE sponge: Shows high absorption capacities for carbon tetrachloride and gasoline oil. Availability and mechanical properties would be important factors. Advantages: (i) High Absorption Capacities: (ii) Availability: sLLDPE is a commonly used polymer with widespread availability. This ensures that the sponges can be easily manufactured from recycled LLDPE and obtained, making them accessible for various applications. (iii) Mechanical Properties: The mechanical properties of sLLDPE, such as flexibility, resilience, and durability, make it suitable for use as a sponge. Its ability to retain its shape and withstand repeated use and compression is advantageous in practical applications. (iv) Regenerability: The regenerability of sLLDPE sponges is easily operated several times and reused with removal oils. Depending on the adsorption process and the specific contaminants, it may be not easy to regenerate the sponge and restore its original absorption capacities fully. This can affect its reusability and overall cost-effectiveness. Limitations: (i) Selectivity: The sLLDPE sponge's high absorption capacities for carbon tetrachloride and gasoline oil may limit its selectivity for other types of oils or organic solvents.

**Table 3:** Summarizes the absorption capacities of different materials for removing oils and organic solvents.

No	Materials	capacity (g/g)	Oil/organic solvents	Limitations	Advancements	Ref
1	Natural wool fibers (NWF), recycled wool based nonwoven material (RWNM)) and Sepiolite	5.56, 5.48, and 0.19	Real oily wastewater	Lower absorption capacities and poor mechanical properties	Availability and low cost	^48^
2	Octadecylamine (ODA)/corn stalk pith (CSP)	33.7–43.6	Soybean oil, diesel, and machine oil	Cost and regenerability	High absorption capacities	^49^
3	Acetylated nettle fibers	23.21 and 18.75	Diesel engine oil and crude oil	Availability, environmental impact, and cost	Good absorption capacities	^50^
4	Dried raw orange peel waste (OP)	3–5	Crude oil, engine oil, and diesel fuel	Low oil and solvent absorption capacity	Use of readily available waste material	^51^
5	leaves (L) and roots (R) of Pistia stratiotes	2.3–2.9	Crude oil	Low absorption capacities	Natural material for crude oil absorption	^52^
6	Styrene-PP	18.91 and 26.36	Diesel and lubricating oil	Availability, cost-effectiveness, regenerability, and mechanical properties	Moderate to high absorption capacities for diesel and lubricating oil	^53^
7	Polydimethylsiloxane (PDMS)–graphene sponges	6.5	Gasoline and canola oil	Complete desorption may not be feasible	High absorption capacities for gasoline and canola oil	^54^
8	Carbon aerogel (CA) / bamboo pulp fibers	50 – 150	Organic solvents/oils	Availability, cost-effectiveness, and regenerability	High absorption capacities for organic solvents and oils	^55^
9	Methyltrimethoxysilane/dodecyltrimethoxysilane (MTMS/DTMS)	7.98–13.4	Chloroform and kerosene	Regenerability and cost effectiveness	Moderate absorption capacities for chloroform and kerosene	^56^
10	Graphite modified by CTAB-KBr/H_3_PO_4_	4.1–7.44	Engine oil, crude oil, diesel oil and gasoline oil	Lower absorption capacity compared to other materials	Adsorption capabilities for engine oil, crude oil, diesel oil, and gasoline oil	^57^
11	Bio-Based Polyurethane	62 and 65	Diesel and gasoline	Cost and regenerability	High absorption capacities for diesel and gasoline	^58^
12	Poly(3-hydroxybutyrate) and Polylactide Polyester	Up to 46.3	Baltic crude oil	Cost and availability	High absorption capacities for Baltic crude oil	^59^
13	Polycaprolactone (PCL)/Poly-D, L-Lactic acid (PDLLA) modified melamine	3.3∼8.7	Disparate viscous oils	low absorption capacities and cost-effectiveness	used for disparate viscous oils	^60^
14	sLLDPE sponge	38.8 – 39	Carbon tetrachloride/gasoline oil	Selectivity	High absorption capacity Availability:manufactured from Recycled LLDPE High Mechanical Properties: Flexibility, resilience, and durability Regenerability Cost effective	Current study

## Conclusion

Effective materials for oil–water pollution removal are vital for environmental protection. A novel method converted recycled LLDPE to oil–water separation sLLDPE sponge using thermal treatment and gamma irradiation (0, 30, 50, 70, 90 kGy). Thermal treatment formed a porous structure, and gamma irradiation crosslinked, enhancing oil removal. The adsorption capacities of the sLLDPE sample irradiated at 50 kGy were higher for various solvents, including non-polar, moderately polar, and polar aprotic solvents. The maximum sorption capacities increased by approximately 43.7% for carbon tetrachloride, 10.2% for 1,2 dichloroethane, 12.38% for diethyl ether, 5.6% for toluene, 3.8% for cyclohexanone, 1.7% for butanone, and 17.1% for n-heptanone, compared to the sample irradiated at 0 kGy. This enhancement can be attributed to the introduction of attachment groups on the sLLDPE surface through radiolytic reactions, leading to increased hydrophobic affinity and the attachment of additional active site groups. Furthermore, the maximum adsorption capacities increased by approximately 216.2% for crude oil, 235.3% for gasoline oil, 24.1% for motor oil, 111.5% for pump oil, and 18.6% for waste oil, compared to the sample irradiated at 0 kGy, attributed to radiolytic reactions increasing hydrophobicity and active sites. Gamma irradiation enhances sLLDPE's environmental remediation potential by boosting adsorption capacities, making it a promising material for oil–water pollution removal. This study’s findings underscored gamma irradiation's effectiveness in enhancing sLLDPE's adsorption capabilities and suggested avenues for further research on optimization and practical application.

### Supplementary Information

Supplementary Video 1.

Supplementary Video 2.

Supplementary Video 3.

Supplementary Video 4.

Supplementary Information 1.

## Data Availability

All data generated or analyzed during this study available from the corresponding author on request.
