# Supplementary material for: Gamma irradiation-enhanced performance of waste LLDPE thermally transformed into advanced sponge-like material for oil decontamination
Source: Sci Rep. 2023 Nov 6;13:19222. doi: 10.1038/s41598-023-46194-w (PMC10628294; doi:10.1038/s41598-023-46194-w)
Supplement: Supplementary file 5 — Supplementary Information 1. [file 41598_2023_46194_MOESM5_ESM.docx]

**Video legends**

Supplementary Video 1. Thermally conversion of (rLLDPE) to sponge-like material.

Supplementary Video 2. The oil-water separation performance of sLLDPE (50 kGy) sponge material by a self-made column.

Supplementary Video 3. The oil-water separation performance of sLLDPE (50 kGy) sponge material by glass funnel.

Supplementary Video 4. Visualization of oil absorption by sLLDPE (50 kGy) sponge.
